# Capnometry-Guided Respiratory Intervention in Veteran PTSD: Impact on Symptom Clusters

**DOI:** 10.3390/healthcare13040390

**Published:** 2025-02-11

**Authors:** Robert N. Cuyler, Juliette S. Mojgani, Julio Cezar Albuquerque da Costa, Rafael C. Freire

**Affiliations:** 1 Freespira, Inc., Houston, TX 77005, USA; 2 School of Medicine, Queen’s University, Kingston, ON K7L 4X3, Canada; 16sjm18@queensu.ca; 3 Institute for Psychology, Federal University of Alagoas, Macelo 57072-900, Brazil; julio.costa@ip.ufal.br; 4 Department of Psychiatry, Queen’s University, Kingston, ON K7L 4X3, Canada; rafaelcrfreire@gmail.com; 5 Centre for Neuroscience Studies, Queen’s University, Kingston, ON K7L 4X3, Canada; 6 Kingston General Hospital Research Institute, Kingston Health Sciences Centre, Kingston, ON K7L 2V7, Canada; 7 Laboratory of Panic and Respiration, Instituto de Psiquiatria, Universidade Federal do Rio de Janeiro (UFRJ), Rio de Janeiro 22290-140, Brazil

**Keywords:** PTSD, trauma, stress disorders, CGRI, Freespira, panic disorder, biofeedback, digital therapeutic, carbon dioxide sensitivity, hyperarousal

## Abstract

**Background/Objectives:** Post-traumatic stress disorder (PTSD) is a challenging psychiatric condition to treat, with suboptimal recovery and difficulty tolerating exposure-based psychotherapies often noted in outcomes research. The aim of this study was to examine patterns of symptom reduction in veterans with PTSD treated with a Capnometry-Guided Respiratory Intervention (CGRI), a 28-day treatment teaching about the normalization of respiratory rate and exhaled carbon dioxide levels via biofeedback. We hypothesized reductions in total PCL-5 scores and all symptom clusters immediately post-treatment but with relative resistance to changes in hyperarousal symptoms, as reported in the outcomes of research using other evidence-based psychotherapies. **Methods:** In this report of real-world outcomes, we included 164 veterans treated with CGRI. Pre- and post-treatment PTSD Checklists for DSM-5 (PCL-5) scales were recorded and analyzed based on the total, cluster, and item scores. Subjects were additionally classified into Recovered, Improved, or Suboptimal subgroups based on their response to treatment. Data were compiled during routine clinical care and are available for retrospective analysis. **Results:** Treatment response was reported in 53% of participants, with a mean total PCL-5 score reduction of 12 points post-treatment (effect size, Glass’s Δ = 0.99, large) and individual PCL-5 clusters showing medium to large effect sizes (effect size = 0.71 to 0.98). Contrary to our hypothesis, a large effect size was found in the hyperarousal cluster, with post-treatment scores being significantly improved compared to pre-treatment scores (effect size = 0.98). In the Recovered group, all 20 PCL-5 items showed significant declines, while significant reductions were reported in some items in the Improved group and no item improvements were noted in the Suboptimal group. **Conclusions:** Consistently with prior published trials reporting overall improvements in PTSD symptoms, in this report, the CGRI produced clinically meaningful reductions in PCL-5 cluster scores in addition to total scores. Unlike reports from several trials of cognitive therapies, this study found hyperarousal symptoms to be responsive to treatment. The CGRI shows evidence of improvement across the range of PTSD symptoms in the immediate post-treatment interval. The absence of an extended post-treatment follow up introduces uncertainty concerning the durability of benefits experienced, although previous CGRI research on both panic disorder and PTSD has shown the maintenance of symptom reduction in six- to twelve-month intervals.

## 1. Introduction

Post-traumatic stress disorder (PTSD) is a life-impairing condition that develops following exposure to significant traumatic events, including combat, violent crime, sexual assault, accidents, and natural disasters. Epidemiological data show a lifetime prevalence of 3.4% to 8.0% among civilians and of 7.7% to 13.4% among U.S. military veterans. Rates for women are approximately twice those for men, while prevalence is higher among subpopulations such as refugees, first responders, Native Americans, and heavy substance users [1]. A significant percentage of affected individuals develop chronic PTSD. In addition, the PTSD diagnosis is associated with an increased risk of medical and psychiatric comorbidities, which, in turn, have a negative impact on response to treatment [2,3,4,5,6].

Evidence-based treatment approaches for PTSD recommended by the National Center for PTSD include several psychotherapies (prolonged exposure, cognitive processing therapy, and Eye Movement Desensitization and Reprocessing) as first-line therapies and antidepressant medications as second-line therapies [7]. Despite the robust evidence bases for the psychotherapies, significant numbers of individuals lack access to these therapies, or have shown substantial dropout rates in both clinical trials and routine clinical use. Tolerability is a challenge for therapies that require the retrieval and processing of traumatic memories, in particular for military-related PTSD [8]. On the other hand, the adherence and tolerability of anti-depressant medications are limited by side effects (e.g., nausea, weight gain, sexual dysfunction, agitation) and length of time before benefits are noticed. Suboptimal adherence rates for individuals with PTSD are noted for both treatment responders and non-responders [9]. These limitations create both an urgency and opportunity for additional treatment approaches that are accessible and tolerable in PTSD.

While a substantial body of the literature has addressed the role of dysfunctional respiration and breathing interventions in panic disorder, evidence of their use in PTSD is sparse. The investigation of a yoga-based breathing intervention (Sudarshan Kriya yoga, or SKY) showed significant benefits in terms of PTSD symptom reduction, startle responses, and hyperarousal symptoms [10]. A follow-up trial found the SKY protocol to be non-inferior to cognitive processing therapy [11]. Further research is required on respiration and breathing interventions, as it may lead to improved tolerability and adherence compared to the high dropout rates and suboptimal outcomes seen in current evidence-based therapies for PTSD.

In this paper, we will present an emerging treatment option for the treatment of PTSD, originally developed as an intervention aimed at normalizing dysfunctional respiration in individuals with panic disorder [12]. Capnometry-guided respiratory intervention (CGRI), commercially available in the form of Freespira^®^, is a prescription digital therapeutic that combines a Class II medical device with a software platform, which has been FDA-cleared for the treatment of panic disorder (2013) and PTSD (2018). The intervention provides breath-to-breath feedback on the respiratory rate and exhaled carbon dioxide levels in a 28-day, at-home course of treatment.

A review of the relevant literature addresses the overlap between panic disorder and PTSD from an epidemiological, physiological, and clinical perspective, particularly the negative impact of panic/PTSD co-morbidity. We will follow this with an analysis of outcomes of veteran treatment completers, with a focus on the impact of this treatment on overall PTSD Checklist for DSM-5 (PCL-5) scores as well as an examination of the changes among the four recognized clusters of PTSD symptoms available from the PCL-5 (B—re-experiencing, C—avoidance, D—negative alterations in cognition and mood, E—hyperarousal) and their component items [13]. Specifically, we will examine whether meaningful differences between cluster scores and individual PCL-5 items for veteran treatment completers differ at pre-treatment and post-treatment intervals. We hypothesize that the CGRI intervention will produce benefits across all four clusters of the PCL-5, but that hyperarousal symptoms will be differentially resistant to change in both treatment responders and non-responders, as has been highlighted in prior studies [14,15].

Panic attacks and panic disorder play an important but insufficiently recognized role in PTSD co-morbidity. In general, the presence of panic attacks and full criteria panic disorder are associated with the onset and persistence of other mental health disorders as well as impaired life function at extended follow-up [16]. A 5-year longitudinal study of youths aged 14–24 with panic attack history at baseline found an increased risk of any anxiety disorder, any mood disorder, and any substance use disorder at follow-up [17].

The specific epidemiological relationship between panic attacks and PTSD is explored by Berenz and colleagues, who examined the temporal associations between the onsets of the two conditions [18]. The authors provide evidence of a bi-directional and highly co-morbid relationship between panic and PTSD, consistent with past studies that link the genetic and environmental risk factors common to the two conditions. A history of panic attacks often pre-dates trauma exposure, while exposure to traumatic events frequently provokes new-onset panic attacks in previously asymptomatic individuals. Rates of current panic attacks are approximately 70% in individuals initiating treatment for PTSD. The authors also highlight the potential for incorporating panic-focused interventions into PTSD treatment in light of the overlapping nature of the two conditions.

The role of panic symptoms in PTSD is explored in a literature review by Teng and colleagues [19]. For individuals with PTSD, heightened vigilance to both bodily and external perceptions of threat create learned associations for subsequent symptom surges, similar to the presence in PD of both cued and un-cued panic attacks. Un-cued panic attacks are widely recognized in panic disorder, but are common in PTSD as well. Cued panic attacks in a PD population include reactions to airplane turbulence, freeway driving, and enclosed spaces. In PTSD, cued symptom surges may be triggered by sights/smells/sounds that have learned associations to traumatic events. An example is an Iraq veteran encountering an overturned garbage can while driving, evoking fear of encountering an improvised explosive device. The authors posit a conditioning-based model—“This model proposes that internal (e.g., emotions, physiological arousal, cognitions) and external (e.g., places, smells, sounds) stimuli experienced during a traumatic event served as conditioned cues that combine to form a fear-based, information processing network … any component of this network that becomes activated will, in turn, activate other components within the network, leading to the experience of anxiety and, possibly, panic symptoms” ([19], p. 269). Additional commonalities include the development of avoidance behaviors as well as substance use serving to numb symptoms.

Evidence of condition overlap from a physiological perspective comes in evidence that both PD and PTSD patients react to carbon dioxide challenge testing [20,21]. The PTSD study found that CO_2_ challenge provoked dissociative symptoms and sudden traumatic memories, as well as panic symptoms. Similarly, Telch and colleagues demonstrated that soldiers’ high reactivity to a single inhalation of CO_2_ at pre-deployment to combat zones was predictive of post-deployment PTSD diagnosis when compared to subjects who were unreactive to challenge [22].

An important area of distinction concerns triggers for symptom surges; individuals with panic disorder generally identify distressing bodily sensations (such as tachycardia or dyspnea) as triggering events for panic attacks, while individuals with PTSD generally identify traumatic memories as triggers for panic attacks [23]. These clinical observations are corroborated by functional imaging studies that suggest that somatic hypersensitivity is characteristic of PD (largely mediated by brainstem pathways), while hyper-alertness to perceived external danger (largely mediated by mid-brain pathways centered on the amygdala) is characteristic of PTSD [24].

The impact of panic on PTSD in veteran populations was addressed in a report from the VA Medical Center’s primary care settings. Panic disorder was significantly more prevalent in this setting than in the general community or community primary care settings, and was found to be highly co-morbid with PTSD. Those individuals with a combination of PD and PTSD showed more severe psychiatric symptoms and higher healthcare utilization compared to those with no psychiatric conditions or with either PD or PTSD [25]. Additional evidence for the negative synergy of these conditions comes in a review of studies examining the impacts of diagnosis on suicide attempt rates, finding that PTSD followed by PD have the strongest associations with self-harm [26].

A meta-analysis of 112 randomized control studies of PTSD treatments provided some general findings of importance. Psychotherapy studies had significantly larger effect sizes compared to medication treatment. For both psychotherapy and medication studies, those trials with more women and fewer veteran participants had larger effect sizes [27].

Despite the heterogenous nature of PTSD symptoms and the presence of a recognized methodology for empirically measuring symptom clusters, the literature examining pre- and/or post-treatment data on symptom clusters is relatively sparse. A study of female veterans treated with two evidence-based psychotherapies (10 weekly visits of prolonged exposure and present centered therapy) provided evidence of clinically meaningful improvement, measured immediately post-treatment. Among the symptom clusters, physiological reactivity, hyperarousal and sleep disturbance symptoms were most resistant to change, even for individuals who no longer met diagnostic criteria for PTSD [14]. These findings mirror results of a study using cognitive processing therapy with active duty service members, which measured PCL-5 symptom clusters immediately post-treatment following the six weeks/twelve session protocol [15]. Residual hyperarousal symptoms remained differentially elevated compared to other symptom clusters across outcome groups classified as recovered (meeting thresholds for a reliable change index as well as clinically significant change), improved (meeting threshold for a reliable change index only) or suboptimal (meeting neither threshold). The authors noted the importance of developing additional treatment approaches that might more robustly address hyperarousal symptoms.

In an additional study of PTSD symptom clusters, Marks and colleagues [28] provided an analysis of civilian emergency dispatchers compared to members of an infantry brigade. Of note is that the dispatchers were repeatedly exposed to indirect traumatic exposure during emergency calls. Dispatchers were more likely than soldiers were to meet DSM-5 criteria for intrusive symptoms (Cluster B) and avoidance symptoms (Cluster C). Soldiers who had been deployed to a combat zone were more likely to meet criteria for hyperarousal symptoms (Cluster E) and to meet full criteria for PTSD as measured by the PCL-5. An item analysis revealed that dispatchers were more likely to engage in cognitive rather than physical avoidance of trauma reminders, which may reflect the nature of indirect traumatic exposure for these individuals, who are taking and coordinating action on calls that include medical emergencies, crime, accidents, domestic violence and the like. As treatment was not provided in this study, no information was available regarding differential responses in cluster scores post-treatment. Reports of treatment outcomes for therapies directed at combined panic and PTSD symptoms are sparse. One example is a report on a pilot study on intensive weekend group treatment of combat veterans. The modality included cognitive restructuring and interoceptive exposure methods, supplemented with psychoeducation. Anxiety sensitivity, posited as a driver of both panic and PTSD symptoms, was a target of the intervention strategy. Large effect sizes were obtained for a range of measures including those for panic frequency, PTSD symptoms, anxiety sensitivity, anxiety, depression, overall well-being, and overall wellbeing [29].

The CGRI intervention is a modification (with a different capnometry device and data capture capability) of the predicate system developed by Meuret and colleagues [12] for the treatment of panic disorder. Published formal clinical trials [30] and outcomes in real world settings [31,32] have reported clinical benefit, favorable side effect profiles and adherence based on the intersection of panic and PTSD symptoms detailed above. Extension to PTSD populations was tested at the Palo Alto V.A. in a clinical trial, including both veterans and civilians, conducted with an open-label design. Participants averaged 19 years pre-enrollment symptom history [33]. Eighty-two percent of subjects showed 13-point or greater reductions in CAPS-5 scores six months post-treatment. The FDA granted clearance for the intervention as an adjunct treatment for PTSD following submission of safety and benefit data from this study. In an analysis of real-world CGRI outcomes by the current author, clinically significant symptom reductions were obtained in 72% of participants classified as PTSD (mean decrease of 20 PCL-5 points) and 65% of participants classified as panic disorder [34]. A follow-up report on PTSD outcomes from two commercial insurance plans (*n* = 208) showed a mean PCL-5 decrease of 24 points (effect size = 1.10) and 81% adherence rates [35]. These two reports on CGRI real-world evidence provide reviews of potential mechanism(s) of action relevant to panic and PTSD symptoms, and are not repeated here.

A randomized control trial [36] using a modification of Meuret’s protocol examined the impact on PTSD hyperarousal symptoms in a veteran population, finding non-significant separation from a wait list control group. The interpretation of these findings is complicated via utilization of a modified protocol when using a single respiratory rate only, and assessing impact on hyperarousal symptoms only. In addition, the study population was characterized by chronicity and severity, with a mean 27-year history since primary trauma and initial CAPS-5 scores averaging 70 points. The relevant co-morbidities include 50% rates of traumatic brain injury and 74% rates of substance use disorder.

In summary, we will examine whether this treatment modality produces narrow or broad-spectrum symptom improvement, as measured by changes across the clusters of the PCL-5. In addition, we hope to determine whether the pattern of resistance to change in hyperarousal symptoms is characteristic of the CGRI intervention, as has been found in prior psychotherapy studies. The initiative to undertake this review of veterans’ treatment outcomes was largely driven by questions from currently referred and prospective V.A. clinicians inquiring as to whether specific symptoms or groups of symptoms were more likely or unlikely to respond to the CGRI intervention.

## 2. Materials and Methods

### 2.1. Sample

This sample comprised 164 U.S. military veterans (mean age = 47, males = 119, females = 45) who began treatment with CGRI between 8 December 2020 and 22 January 2024. The study assessed outcome characteristics of treatment completers classified at intake as PTSD, thus only those with pre- and post-treatment PCL-5 scores recorded in the secure database were included in the analysis. Patients with pre-treatment PCL-5 scores considered below the threshold (total score < 28) were excluded from the analysis. PTSD diagnosis was not independently verified before initiating treatment, and therefore, the identification of a Criteria A primary trauma was the responsibility of the referring clinician, and was not recorded in this dataset. Therefore, the sample cannot be separated into combat vs. non-combat categories, nor are history of sexual assault or other trauma sources available for analysis.

The referring/treating V.A. clinician provided authorization for treatment, following FDA guidelines. Following referral, screening consisted of a brief health history to determine the presence of potential contraindications. As per FDA stipulations, individuals who were pregnant or diagnosed with Obesity Syndrome of Hypoventilation were excluded from enrollment. Additionally, individuals with Chronic Obstructive Pulmonary Disease or other advanced respiratory illness characterized by lung damage and abnormally high end-tidal carbon dioxide levels were excluded, as the intervention is principally aimed at raising and stabilizing low to normal carbon dioxide levels. A medical clearance from a personal physician was obtained if the health history revealed medical complexity, such as unstable seizure disorder or asthma. Individuals with active suicidal ideation, schizophrenia, or active psychosis were screened and the referring clinician was notified, reflecting the need for reasonable current stability and likely ability to engage consistently with an at-home intervention. A pre-treatment PCL-5 was obtained for all participants at enrollment.

The data source for the study was a secure database compiling de-identified adherence and symptom scale scores. Inclusion was limited to individuals recorded as V.A. beneficiaries. Participants signed a terms and conditions form, which included consent for de-identified data use in research. The Institutional Review Board at the University of Texas Austin reviewed a previous retrospective review of de-identified data (IRB ID-STUDY00003542) and granted IRB exemption [34], and thus was not sought again for this project.

### 2.2. Procedure

**Figure 1:**
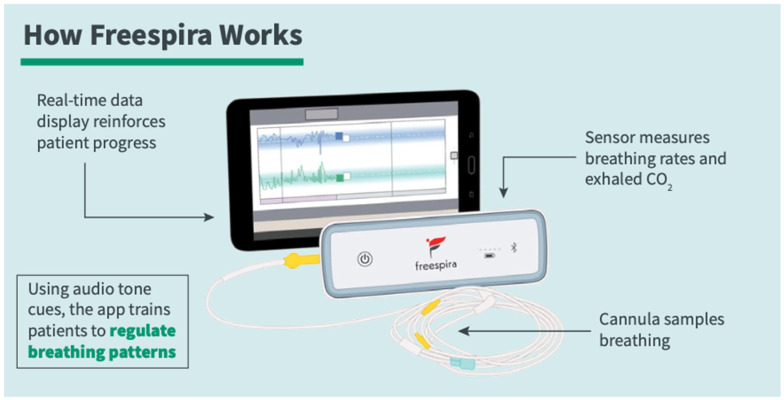
Freespira CGRI system.

The CGRI intervention [34,35] provides feedback on respiratory style (respiratory rate (RR)) and end-tidal carbon dioxide levels (etCO_2_), aimed at normalizing dysfunctional breathing. This Class II medical device consists of a respiratory sensor sampling breathing via a nasal cannula, linked via Bluetooth to a tablet computer displaying RR and etCO_2_. Sessions are of 17 min duration, with recommended use twice daily for 28 days (see Figure 1).

**Figure 2:**
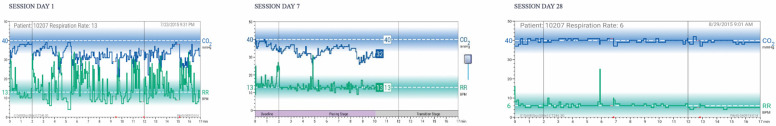
Respiratory graph example.

The seventeen-minute session has three phases—(a) a two-minute baseline measurement, recording respiratory characteristics only; (b) a ten-minute pacing phase, in which rising and falling audio tones guide inhalation and exhalation, users view graphs of RR and etCO_2_ that guide respiratory targets, and instructions given via the tablet computer guide users to modify air intake to move and hold etCO_2_ values around 40 mmHg; and (c) a five-minute transition phase in which audio tones are withdrawn—the purpose of this phase is to promote the self-management of respiratory stability with limited feedback; see Figure 2 for an example of respiratory graphs spanning the 28-day treatment episode. The target respiratory rate is decreased weekly (13 to 11 to 9 to 6 RR). The automatic upload (via Wi-Fi) of session data to a secure server occurs at the end of each session.

Following the obtaining of participants’ referral for treatment and pre-treatment PCL-5, an initial 45 min secure video teleconference was conducted with an assigned health coach. These personnel are full- or part-time employees with backgrounds as master’s level social workers, licensed professional counselors, or individuals with formal health coaching backgrounds. The job role specifically limits the coaches to training, education on principles of optimal breathing style, and support/encouragement. The activities center on the following: (a) explanation of the treatment rationale, (b) determination of patient goals/expectations, (c) education regarding diaphragmatic breathing and respiratory targets, (d) instructions for using the sensor/tablet, and (e) observation/feedback while the patient undertakes an initial session.

Weekly 10–15 min follow-up sessions with the health coach reviewed the prior week’s sessions (available to the patient on the tablet and the coach on a secure portal) and provided coaching for continued progress. Indications of suboptimal adherence prompted communication between visits to determine reasons and to provide encouragement in the recommended session schedule. Final PCL-5 scales were obtained within days before or after the final session. Although interactive video is the preferred method for these follow-up sessions, communication via phone or text can be substituted based on patient preference. Weekly coaching notes are reviewed by clinical management and an end-of-treatment summary report consisting of initial and final session graphs, adherence information, coach observations, and symptom changes during treatment is sent to the referring clinician. The secure database provides a source of metrics for research such as this, as well as internal quality improvement and summary reports for client organizations.

### 2.3. Measures

The 20-item PCL-5 was used as a self-report measure of PTSD symptoms. The PCL-5 is a 20-item self-report scale based on DSM-5 criteria. The items are endorsed by participants from 0 (Not at all) to 4 (Extremely), and total scores range from 0 to 80 [37]. As per recommendations from the National Center for PTSD, a 10-point reduction in pre- to post-treatment scores was chosen as a minimum threshold for clinically meaningful change. The scale is recommended for both inclusion in routine clinical practice as well as in research settings, particularly where access to a structured clinical interview such as the Clinician-Administered PTSD Scale (CAPS-5) is not feasible. In this study, the scale was administered “Without Criteria A”, or with instructions and items only, as referring clinicians were responsible for assessment and referral based on the existence of a PTSD diagnosis. The examination of the psychometric properties of the scale shows strong evidence for reliability and validity [38].

Means and standard deviations of pre- and post-treatment scores were recorded for total PCL-5 scores as well as for the four recognized clusters of PTSD symptoms (cluster B—re-experiencing, cluster C—avoidance, cluster D—negative alterations in cognition/mood, and cluster E—alterations in arousal/reactivity). Percent reductions in symptoms for total and cluster scores were obtained. Patient symptom scale data and physiological metrics were uploaded and maintained in a secure company server. Breath-to-breath and session mean respiratory values were recorded, but these metrics were not a focus of this analysis.

The examination of the obtained data had a number of targets: (a) pre-treatment symptom severity difference (in total and cluster scores) for the total population as well as by gender; (b) post-treatment total and cluster symptom reductions, and (c) analyses of changes in post-treatment item and cluster scores in order to determine whether the clinical benefit of CGRI was narrow or broad-spectrum, and whether hyperarousal remained differentially resistant to change among the four PCL-5 clusters.

### 2.4. Statistical Methods

Changes in mean PCL-5 total and cluster scores were evaluated via *t*-test, with 95% confidence intervals and effect size calculated. Treatment adherence was recorded as number of completed sessions, with percent adherence calculated as the number of completed sessions divided by 56, the recommended treatment “dosage”. The completion of more than 56 sessions was coded as 100%. Effect sizes for samples with equal sample sizes were calculated with Cohen’s d, while Glass’s d was calculated for unequal sample sizes.

Work by Miles et al. on PCL-5 symptom cluster changes following cognitive processing therapy (CPT) [15] provided both a methodological framework and a basis for comparison concerning the impacts of CGRI on PTSD symptoms. Calculated using this methodology [39], the reliable change index (RCI) for this sample of 164 veterans treated with CGRI was 10.34, equivalent to recommendations [13] of a 10-point reduction as clinically meaningful. The calculation of clinically significant change (CSC) (post-treatment scores at least two standard deviations below the pre-treatment mean of participants) yielded a threshold of a final score of ≤30. The use of these criteria classified participants as follows: “Recovered”, defined as a ≥10-point reduction in PCL-5 scores plus final score ≤30; “Improved”, defined as a ≥10-point reduction in PCL-5 scores; “Suboptimal”, defined as meeting neither RCI nor CSC. Scores marked by participants as elevated at moderate or higher levels (item score 2 on the 0–4 scale) were recorded and mean percentages were calculated, showing the pre- and post-treatment prevalence of PCL-5 items and clusters.

From this, Generalized Estimating Equations (GEE) models were used to comparatively analyze the groups (Suboptimal, Improved and Recovery) through the measurement moments. To this end, the logit link function and binomial distribution were used [37,38]. Furthermore, the odds ratios (ORs) were analyzed, in which values above 1.0 indicated that the analyzed group had a greater chance of presenting the outcome when compared to the reference group, while values below 1.0 showed a decrease in these chances. Furthermore, the association of gender and age with the subject grouping variable was verified, using the Analysis of Variance (ANOVA) and the chi-square test.

## 3. Results

### 3.1. Demographics & PCL-5 Reductions

The mean age of the 164 veterans was 47.36 years. The mean adherence for the total sample was 75% (41.8 completed sessions). The mean PCL-5 score was 55.34 (sd = 12.34) at pre-treatment and 43.13 (sd = 19.46) at post-treatment (*p* < 0.001). This 12.21 mean difference yields an effect size (Glass’s Δ) of 0.99 (Large). Contrary to the hypothesis derived from several reports on cognitive therapies, the hyperarousal cluster (cluster E) showed a large effect size (0.98) equivalent to the total score effect size (0.99) for the total population. Fifty three percent (*n* = 87) of subjects achieved a 10-point or greater reduction in PCL-5 scores. The calculation of PCL-5 cluster scores yielded Glass’s Δ effect sizes and *t*-test *p* values as follows: cluster B = 0.79 (medium) (*p* < 0.001); cluster C = 0.85 (large) (*p* < 0.001); cluster D = 0.71 (medium) (*p* < 0.001); cluster E = 0.98 (large) (*p* < 0.001). Percent symptom reduction across total and cluster scores remained in a tight range of 21.05% (cluster B) to 23.88% (cluster E). Demographics and pre-/post-treatment metrics are shown in Table 1.

### 3.2. Gender Differences

The mean age of male participants (*n* = 119) was 48.67 years, and it was 44.42 for females (*n* = 45). Adherence for male participants was 76% (mean = 42.44 sessions) and it was 72% (mean = 40.36 sessions) for females. The mean total pre-treatment PCL-5 score for females (mean = 57.36, sd = 12.40) was higher than that for males (mean = 54.35, sd = 12.51). Effect sizes for symptom reduction across the symptom clusters for female veterans (range 0.80 to 1.18) were consistently higher than those for male veterans (range 0.67 to 0.91). Sixty two percent of female participants (n = 45) achieved a treatment response (>10-point reduction), compared to fifty percent of male participants (n = 119).

### 3.3. Subgroup Analysis

Pre-treatment symptom severity in the Recovered group (*n* = 42, PCL-5 total $\bar{X}$ = 47.86, sd = 12.61) was notably lower than in the Improved (*n* = 45, $\bar{X}$ = 60.44, sd = 8.99) and Suboptimal (*n* = 77, $\bar{X}$ = 56.84, sd = 12.34) groups. When pre-/post-treatment differences in total PCL-5 scores were calculated, the results were as follows: Recovered, effect size = 2.72 (large); Improved, effect size = 1.91 (large); Suboptimal, effect size = 0.01 (trivial); see Table 2.

When measuring treatment response via the Generalized Estimating Equations (GEE) approach [40,41] for total PCL-5 score and clusters, significant reductions were obtained for all total PCL-5 scores and all clusters in the Recovered group; for the Improved group, only the second cluster (Avoidance) failed to show statistically significant improvement. Finally, for the Suboptimal group, no statistically significant changes emerged for total or cluster scores. When examining the consistency of cluster scores within the subgroups, no significant differential results were found within the subgroups (Suboptimal, Improved and Recovered), with the exception of cluster C (Avoidance) in the Improved group. The intervention demonstrated effectiveness across all 20 PCL-5 items in the Recovered group, indicating that the treatment was successful in addressing the full spectrum of PTSD symptoms used in the scale. For the Improved group, a significant effect was noted for items Q1 (intrusions), Q2 (nightmares), Q3 (flashbacks), Q4 (emotional distress), Q5 (physical reactivity), Q11 (strong negative emotions), Q15 (irritability) and Q17 (hypervigilance). No treatment effect was observed in the Suboptimal group at the PCL-5 item level as a result of the intervention; see Table 3.

Following this, Generalized Estimating Equations (GEE) models were used to comparatively analyze the groups (Suboptimal, Improved and Recovery) through the measurement moments. To this end, the logit link function and binomial distribution were used [37,38]. Furthermore, the Odds Ratios (OR) were analyzed, in which values above 1.0 indicated that the analyzed group had a greater chance of presenting the outcome when compared to the reference group, while values below 1.0 showed a decrease in chances. Furthermore, the association of gender and age with the subject grouping variable was verified, using the Analysis of Variance (ANOVA) and the chi-square test. Additional information from the GEE analysis is available in Table 4 and Table 5.

**Figure 3:**
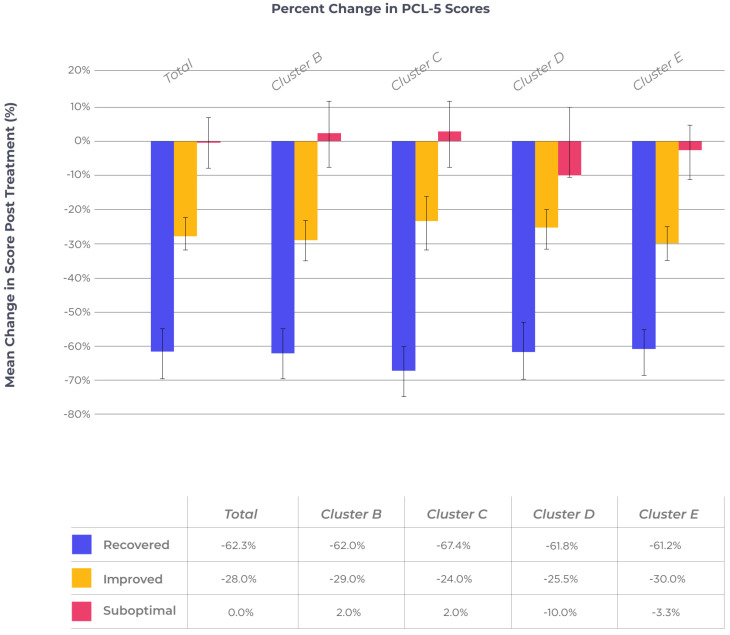
Pre–post-treatment percent change in PCL-5 scores across treatment response subgroups.

See Figure 3 for a graphic display of percentage change (with 95% confidence intervals [40]) for total and cluster scores.

Finally, the association of subjects’ classifications with gender and age were investigated. The results indicate that there were no associations between groupings when performed by gender (χ^2^(2) = 4.955, *p* = 0.084) and by age (F(2) = 1.095, *p* = 0.337, η^2^ = 0.013).

## 4. Discussion

### 4.1. Outcome Interpretation

This analysis of real-world evidence is best characterized as a pragmatic trial, based on observations derived from actual clinical practice under usual conditions [42]. For this group of veterans completing CGRI treatment, an overall large effect size was obtained, as well as clinically meaningful reductions in PTSD symptoms for a majority of participants (53%), with ≥10-point reductions in PCL-5 scores (a threshold recommended for the interpretation of this scale [7]) as well as the calculated value of the Reliable Change Index for this sample. Forty-seven percent of participants (n = 77) did not meet this threshold immediately post-treatment. As has been reported elsewhere [43], female participants showed greater improvements than males, with greater effect sizes for overall and all cluster scores.

Regarding the primary hypothesis tested here, the reductions in hyperarousal symptoms overall and for two of the subgroups (Recovered and Improved) were clinically significant. This finding suggests a potentially different mechanism of action for the CGRI treatment compared to cognitive therapies, where treatment resistance for hyperarousal symptoms even in treatment responders has been noted [14,15]. The CGRI intervention can be reasonably termed a “bottom-up” therapy [44], with a focus on attending to and normalizing bodily (respiratory) function via physiological feedback, thus aiming directly at reducing autonomic reactivity. No inquiries into traumatic exposure or thoughts/emotions associated with trauma history are made by the assigned health coaches, thus avoiding an integral feature of exposure-based therapies often implicated as an impediment to adherence. The results also demonstrate consistent impacts across the range of PCL-5 symptom clusters.

Of note, the Recovered cohort began treatment with lower total PCL-5 scores than the Improved and Suboptimal groups (total $\bar{X}$ = 48 points vs. 60 and 56, respectively), but also substantially greater post-treatment score reductions (30 points vs. 17 and 0, respectively). These results suggest that symptom severity may serve as one variable with potential use in identifying optimal candidates for the inclusion of this intervention into their treatment plans. The presence of suboptimal outcomes in 47% of participants mirrors evidence of treatment resistance in PTSD, particularly in individuals with greater symptom severity, psychiatric comorbidity, poor physical health, male gender, and early life and/or multiple trauma exposures [45]. It was notable that participants in the Improved and Suboptimal groups began treatment with equivalent overall severity scores, as measured by the PCL-5. Treatment adherence did not differ significantly among the three subgroups (although it was marginally higher in the Recovered group), and was unlikely to play a role in the differential outcomes observed here.

The separation of the participants into cohorts based on the extent of symptom reduction gives a useful perspective, one of which is to remind ourselves that while total group effect sizes are important in quantifying the clinically meaningful impacts of an intervention, we do not treat average patients with average outcomes. As noted above, participants in the “Recovered” group began treatment with lower PCL-5 scores and greater symptom reduction according to total PCL-5 scores, but they also attained significant reductions across all four symptom clusters and each of the twenty scale items. In the Improved group, the overall symptom improvement was significant, but the change in the Avoidance cluster failed to show significance. By contrast, the Suboptimal group recorded no change at the total, cluster or item levels. The future refinement of the CGRI intervention may include detailed assessment of non-response to prior therapy, chronicity and co-morbidity to determine if predictors of treatment response can be identified.

The finding that hyperarousal/hypervigilance is responsive to the intervention suggests the potential merit of staging this treatment prior to engagement in exposure-focused methods for individuals with a high risk of dropout or prior non-response to psychotherapy. Additionally, significant improvements in PCL-5 Question 2 (nightmares) in both the Recovered and Improved subgroups and Question 20 (insomnia) in the Recovered subgroup are noteworthy, since impaired sleep quality is both characteristic of PTSD and resistant to change with psychotherapy [46].

In the Improved cohort, it was notable that the impact on the Avoidance cluster was non-significant, in contrast to the other clusters. One possible explanation for this finding is that symptom types may change at different rates, and that improvement in other realms may be necessary (for example, reduced intrusive trauma memories and/or hyperarousal) before avoidance behaviors begin to alter, leading to the possibility that changes in avoidance behavior will not accrue within the 28-day span of this treatment, but may emerge later. In Ostacher’s open-label CGRI trial [33], cluster scores were not available, but total CAPS-5 scores showed incremental additional decreases from immediately post-treatment ($\bar{X}$ = 31.8) to six-month follow-up ($\bar{X}$ = 26.2), suggesting that a trajectory of additional improvement may be notable after the formal treatment episode had ended.

As noted in the introduction, research on utilizing treatment methods developed for panic disorder in PTSD populations is limited but promising. Teng and colleagues worked [29] with group treatments for veterans with PTSD using psychoeducation and interoceptive exposure methods, showing a broad range of improvement across both panic and PTSD symptoms. This suggests that interoceptive and CGRI methods (which both primarily focus on panic symptoms) may serve as a “gateway” approach to treating PTSD that has the potential to bypass the tolerability hurdles that compromise adoption and adherence for therapies that include the required retrieval of traumatic reminders.

From a more subjective standpoint, the lead author can reflect on his overview of several thousand completed CGRI treatments, which include reviews of physiologic metrics, symptom scale scores, and observations by health coaches summarizing their care of participants at the end of treatment. One common theme seen reflected in the study participants was the use of learned self-management skills. Participants described “trying out” engaging in previously avoided activities and situations, finding that these situations were now more manageable. Another is the reduction in the frequency and intensity of nightmares and an increased ability to resume sleep after using the recommended breathing skills. Reports of decreased irritability included comments on “taking a few breaths”, thereby moderating immediate or impulsive responses to perceived irritants or provocations. Another measure of improvement is reflected in comments that spouses and family find the participant calmer, less reactive, and “easier to live with”.

Pragmatic trials such as this provide observational data and do not further the understanding of mechanism of action for this intervention. The replication of these findings with a randomized design, meaningful control conditions, and more extensive evaluations of pre-treatment characteristics is a vital next step, particularly concerning the impacts on hyperarousal symptoms. Future research initiatives, which include grant proposals currently in process, will incorporate a randomized design and a broader range of outcomes, including measures of sleep quality, irritability, quality of life, and additional continuous physiological monitoring. The evaluation of a combined treatment with CGRI and prolonged exposure and/or cognitive processing therapies has the potential to be used in assessing potential synergistic benefits.

### 4.2. Limitations

Real-world evidence studies such as these have limitations worthy of note. Internal validity concerns include a risk of selection bias and regression to the mean. Interpretations of these results are limited by the use of real-world clinical outcome data lacking a control group and follow-up beyond the immediate post-treatment interval. On the other hand, reports such as these are useful in supplementing formal clinical trials characterized by smaller sample sizes and more restrictive inclusion criteria. Evidence of intervention benefits in ordinary clinical practice is vital in determining the ongoing utility of treatment methods first established in formal trials. Prior trials using CGRI for panic disorder and PTSD showed the durability of post-treatment improvement (without access to the intervention) up to one year post-treatment in PD and six months post-treatment in PTSD [12,30,33]; however, data for determining extended benefits were not available in this dataset.

Compared to formal clinical trials, the real-world nature of this study limits the baseline characteristics of the participants, and may have introduced selection bias due to the referral decisions made by individual clinicians. Referral from the treating VA clinician did not include the specification of an index trauma (single, multiple, combat, sexual assault, or other); therefore, the DSM-5 diagnostic criteria were not independently verified. Documentation of prior or current treatment history was not available. The rationale for the referral (and therefore selection bias) was also not known, which may include clinician readiness or reluctance to adopt new treatment methods, patient failure to respond to previous first- or second-rank therapies, patient preference for a non-pharmacologic option, and reluctance to engage in verbal psychotherapies. Relevant co-morbidities were not known, which likely included a variety of conditions including other psychiatric disorders, traumatic brain injury, substance use disorders, and medical conditions. Another important uncertainty was the extent to which non-responders did not have clinically meaningful panic symptoms pre-treatment, and instead may have had conditions more weighted towards guilt, moral injury, and mood disturbance.

The limitations discussed here also provide an opportunity for the expansion of collaboration with referring V.A. clinicians in order to better understand their reasons for referral and the rationale for including this intervention in individual treatment plans. Such efforts may provide important insights into the improved selection of candidates for this treatment.

## 5. Conclusions

This report is one of a series of analyses of real-world evidence that have evaluated the effectiveness of the CGRI intervention in civilian and now veteran populations. The decreases seen in the PTSD symptoms of this sample of veterans met thresholds for clinically significant change using the CGRI treatment, but these reductions were lower than those obtained in prior studies using civilian samples. The outcomes were positive for PCL-5 total and symptom clusters, including hyperarousal/hypervigilance symptoms that have been reported as resistant to change in psychotherapy trials, even in treatment responders. When participants were stratified into groups based on response to treatment, the sample was separated into pools of individuals showing significant benefits across all (Recovered group) or most (Improved group) measures, including specific items related to nightmares and insomnia. Nearly half of the veterans were classified as having Suboptimal outcomes, with no significant reductions across any of the measurements, emphasizing again the regrettable persistence of symptoms in too many affected veterans. Future efforts to determining the characteristics of responders vs. non-responders would add significantly to efforts to position the CGRI intervention optimally among treatment options. The results also support the value of incorporating panic-focused modalities into the treatment of PTSD. The challenges of treating PTSD remain significant, and broadening the availability of evidence-based treatment options remains an urgent need.

## Figures and Tables

**Table 1:** Pre- and post-treatment outcomes in a sample of veterans.

	Veteran Total	Veteran Male	Veteran Female
	(*n* = 164)	(*n* = 119)	(*n* = 45)
Mean Age (SD)	47.36 (12.28)	48.67 (12.28)	44.42 (11.34)
Mean # Sessions * (SD)	41.80 (13.21)	42.44 (13.19)	40.36 (12.71)
% Adherence **	75%	76%	72%
Treatment Responders *** (% of sample)	n = 88 (53%)	n = 60 (50%)	n = 28 (62%)
PCL-5	Mean PCL-5 score (SD)	Mean PCL-5 score (SD)	Mean PCL-5 score (SD)
Total pre score	55.34 (12.34)	54.35 (12.51)	57.36 (12.40)
Total post score	43.13 (19.46)	43.24 (20.05)	42.71 (18.19)
Score reduction	12.21 (15.21)	11.29 (15.55)	14.64 (13.93)
% Reduction	22.12%	19.46%	26.40%
Effect size (Glass’s Δ)	0.99	0.89	1.18
CL B pre score	13.58 (3.62)	13.21 (3.51)	14.44 (3.89)
CL B post score	10.73 (5.28)	10.63 (5.29)	10.84 (5.39)
% Reduction	21.05%	19.81%	25.43%
Effect size (Glass’s Δ)	0.79	0.73	0.92
CL C pre score	6.16 (1.62)	5.96 (1.67)	6.58 (1.59)
CL C post score	4.78 (2.38)	4.69 (2.48)	4.98 (2.09)
% Reduction	22.19%	18.73%	20.53%
Effect size (Glass’s Δ)	0.85	0.76	1.01
CL D pre score	18.90 (5.61)	18.60 (5.53)	19.44 (6.08)
CL D post score	14.91 (7.07)	14.92 (7.30)	14.56 (6.78)
% Reduction	21.30%	18.55%	23.83%
Effect size (Glass’s Δ)	0.71	0.67	0.8
CL E pre score	16.70 (4.07)	16.59 (4.13)	16.89 (3.97)
CL E post score	12.71 (5.90)	12.82 (6.03)	12.33 (5.55)
% Reduction	23.88%	23.58%	27.60%
Effect size (Glass’s Δ)	0.98	0.91	1.15

Note: CL = cluster; PCL-5 = Posttraumatic Stress Disorder Checklist for DSM-5. * Mean number of sessions attended. ** Percent of sessions completed out of total 56 sessions. *** Treatment response was defined as a >10-point reduction in PCL-5.

**Table 2:** Subgroup analysis for PCL-5 total and cluster scores.

PCL-5 Score	Recovered (SD) *n* = 42	Improved (SD) *n* = 45	Suboptimal (SD) *n* = 77
Pre-Tx Total	$\bar{X}$ = 47.86 (12.61)	$\bar{X}$ = 60.44 (8.99)	$\bar{X}$ = 56.84 (12.34)
Post-Tx Total	$\bar{X}$ = 18.05 (9.02)	$\bar{X}$ = 43.67 (8.52)	$\bar{X}$ = 56.74 (13.83)
% Change	−62.3%	−28.0%	0%
U95CI	−55.8	−23.1	8.3
L95CI	−68.8	−32.3	−8.5
Cohen’s d	2.72 (large)	1.91 (large)	0.01 (Trivial)
Cl B Pre-Tx	$\bar{X}$ = 11.33 (3.15)	$\bar{X}$ = 15.00 (2.37)	$\bar{X}$ = 14.06 (3.86)
Cl B Post-Tx	$\bar{X}$ = 4.31 (2.43)	$\bar{X}$ = 10.58 (2.68)	$\bar{X}$ = 14.39 (4.02)
% Change	−62.0%	−29.0%	2%
U95CI	−54.7	−24.0	12.7
L95CI	−69.2	−34.9	−8.0
Cohen’s d	2.50 (Large)	1.75 (Large)	0.08 (Trivial)
Cl C Pre-Tx	$\bar{X}$ = 5.55 (1.58)	$\bar{X}$ = 6.40 (1.70)	$\bar{X}$ = 6.39 (1.49)
Cl C Post-Tx	$\bar{X}$ = 1.81 (1.33)	$\bar{X}$ = 4.87 (1.49)	$\bar{X}$ = 6.38 (1.57)
% Change	−67.4%	−24%	2%
U95CI	−59.6	−16.0	12.7
L95CI	−75.2	−31.9	−8.0
Cohen’s d	2.56 (Large)	0.96 (Large)	0.01 (Trivial)
Cl D Pre-Tx	$\bar{X}$ = 16.50 (5.85)	$\bar{X}$ = 21.02 (4.28)	$\bar{X}$ = 19.13 (5.56)
Cl D Post-Tx	$\bar{X}$ = 6.31 (3.82)	$\bar{X}$ = 15.60 (3.71)	$\bar{X}$ = 19.29 (5.56)
% Change	−61.8%	−25.8%	−0.10%
U95CI	−53.6	−19.8	10.4
L95CI	−69.9	−31.8	−10.6
Cohen’s d	2.06 (Large)	1.35 (Large)	0.03 (Trivial)
Cl E Pre-Tx	$\bar{X}$ = 14.48 (4.23)	$\bar{X}$ = 18.02 (3.05)	$\bar{X}$ = 17.26 (3.93)
Cl E Post-Tx	$\bar{X}$ = 5.62 (3.26)	$\bar{X}$ = 12.62 (2.95)	$\bar{X}$ = 16.69 (4.50)
% Change	−61.2%	−30.0%	−3.3%
U95CI	−54.7	−24.8	5.5
L95CI	−67.7	−35.2	−12.1
Cohen’s d	2.35 (Large)	1.80 (Large)	0.13 (Trivial)
Adherence $\bar{X}$ (SD)	44.74 (11.20)	40.51 (13.22)	40.96 (13.91)
Adherence Mean Difference		Recovered vs. Improved *p* = 0.11 (NS)	Recovered vs. Suboptimal *p* = 0.13 (NS)

Note: $\bar{X}$ = mean, SD = standard deviation, CI = confidence interval, NS = non-significant.

**Table 3:** Pre- to post-treatment reduction in PCL-5 items across subgroups.

PCL-5 Item	Recovered Subgroup *n* = 42	Improved Subgroup *n* = 45	Suboptimal Subgroup *n* = 77
Intrusions	*p* < 0.001	*p* < 0.001	NS
Nightmares	*p* < 0.001	*p* < 0.002	NS
Flashbacks	*p* < 0.001	*p* < 0.021	NS
Emotional distress	*p* < 0.001	*p* < 0.001	NS
Physical reactivity	*p* < 0.001	*p* < 0.001	NS
REEXPERIENCING CLUSTER (B)	*p* < 0.001	*p* < 0.003	NS
Avoid thoughts	*p* < 0.001	NS	NS
Avoid activities	*p* < 0.001	NS	NS
AVOIDANCE CLUSTER (C)	*p* < 0.001	NS	NS
Inability to recall	*p* < 0.001	NS	NS
Negative cognitions	*p* < 0.001	NS	NS
Self/other blame	*p* < 0.001	NS	NS
Strong negative emotions	*p* < 0.001	*p* < 0.001	NS
Anhedonia	*p* < 0.001	NS	NS
Detachment	*p* < 0.001	NS	NS
Numbing	*p* < 0.001	NS	NS
NEGATIVE ALTERATIONS CLUSTER (D)	*p* < 0.001	*p* < 0.006	NS
Irritability/aggression	*p* < 0.001	*p* < 0.001	NS
Impulsivity	*p* < 0.002	NS	NS
Hypervigilance	*p* < 0.001	*p* < 0.001	NS
Startled	*p* < 0.001	NS	NS
Difficulty concentrating	*p* < 0.001	NS	NS
Insomnia	*p* < 0.001	NS	NS
HYPERAROUSAL CLUSTER (E)	*p* < 0.001	*p* < 0.001	NS
Total score	*p* < 0.001	*p* < 0.001	NS

Note: PCL-5 = Posttraumatic Stress Disorder Checklist for DSM-5; NS = nonsignificant.

**Table 4:** GEE results.

PCL-5 Item	Recovered Subgroup	Improved Subgroup	Suboptimal Subgroup
	*n* = 42	*n* = 45	*n* = 77
Intrusions	χ^2^(1) = 35.584	χ^2^(1) = 15,066.117	χ^2^(1) = 0.066
	*p* < 0.001	*p* < 0.001	*p* = 0.797
Nightmares	χ^2^(1) = 19.033	χ^2^(1) = 9.864	χ^2^(1) = 0.185
	*p* < 0.001	*p* = 0.002	*p* = 0.667
Flashbacks	χ^2^(1) = 14.825	χ^2^(1) = 5.319	χ^2^(1) = 0.694
	*p* < 0.001	*p* = 0.021	*p* = 0.405
Emotional distress	χ^2^(1) = 34.723	χ^2^(1) = 3464.821	χ^2^(1) = 0.118
	*p* < 0.001	*p* < 0.001	*p* = 0.732
Physical reactivity	χ^2^(1) = 28.668	χ^2^(1) = 15,086.854	χ^2^(1) = 0.931
	*p* < 0.001	*p* < 0.001	*p* = 0.335
REEXPERIENCING CLUSTER (B)	χ^2^(1) = 14,725.947	χ^2^(1) = 9.085	χ^2^(1) = 0.060
	*p* < 0.001	*p* = 0.003	*p* = 0.807
Avoid thoughts	χ^2^(1) = 16,640.522	χ^2^(1) = 0.445	χ^2^(1) = 1.917
	*p* < 0.001	*p* = 0.505	*p* = 0.166
Avoid activities	χ^2^(1) = 28.448	χ^2^(1) = 0.209	χ^2^(1) = 1.594
	*p* < 0.001	*p* = 0.648	*p* = 0.207
AVOIDANCE CLUSTER (C)	χ^2^(1) = 33.290	χ^2^(1) = 1.071	χ^2^(1) = 0.517
	*p* < 0.001	*p* = 0.301	*p* = 0.472
Inability to recall	χ^2^(1) = 20.566	χ^2^(1) = 1.115	χ^2^(1) = 0.421
	*p* < 0.001	*p* = 0.291	*p* = 0.516
Negative cognitions	χ^2^(1) = 12.984	χ^2^(1) = 0.381	χ^2^(1) = 0.493
	*p* < 0.001	*p* = 0.537	*p* = 0.482
Self/other blame	χ^2^(1) = 25.178	χ^2^(1) = 1.933	χ^2^(1) = 1.295
	*p* < 0.001	*p* = 0.164	*p* = 0.255
Strong negative emotions	χ^2^(1) = 12.951	χ^2^(1) = 9356.763	χ^2^(1) = 0.000
	*p* < 0.001	*p* < 0.001	*p* = 1.000
Anhedonia	χ^2^(1) = 23.848	χ^2^(1) = 2.329	χ^2^(1) = 0.066
	*p* < 0.001	*p* = 0.127	*p* = 0.797
Detachment	χ^2^(1) = 23.757	χ^2^(1) = 0.209	χ^2^(1) = 0.000
	*p* < 0.001	*p* = 0.648	*p* = 1.000
Numbing	χ^2^(1) = 25.178	χ^2^(1) = 3.164	χ^2^(1) = 0.060
	*p* < 0.001	*p* = 0.075	*p* = 0.807
NEGATIVE ALTERATIONS CLUSTER (D)	χ^2^(1) = 14,148.891	χ^2^(1) = 7.439	χ^2^(1) = 0.000
	*p* < 0.001	*p* = 0.006	*p* = 1.000
Irritability/aggression	χ^2^(1) = 23.850	χ^2^(1) = 12.064	χ^2^(1) = 3.834
	*p* < 0.001	*p* = 0.001	*p* = 0.050
Impulsivity	χ^2^(1) = 9.860	χ^2^(1) = 3.246	χ^2^(1) = 0.000
	*p* = 0.002	*p* = 0.072	*p* = 1.000
Hypervigilance	χ^2^(1) = 20.232	χ^2^(1) = 3562.066	χ^2^(1) = 0.204
	*p* < 0.001	*p* < 0.001	*p* = 0.652
Startled	χ^2^(1) = 19.020	χ^2^(1) = 3.674	χ^2^(1) = 0.118
	*p* < 0.001	*p* = 0.055	*p* = 0.732
Difficulty concentrating	χ^2^(1) = 17.258	χ^2^(1) = 3.010	χ^2^(1) = 0.743
	*p* < 0.001	*p* = 0.083	*p* = 0.389
Insomnia	χ^2^(1) = 23.572	χ^2^(1) = 1.306	χ^2^(1) = 1.260
	*p* < 0.001	*p* = 0.253	*p* = 0.262
HYPERAROUSAL CLUSTER (E)	χ^2^(1) = 17.955	χ^2^(1) = 15,581.933	χ^2^(1) = 0.908
	*p* < 0.001	*p* < 0.001	*p* = 0.341
Total score	χ^2^(1) = 15,222.332	χ^2^(1) = 16,026.728	χ^2^(1) = 0.229
	*p* < 0.001	*p* < 0.001	*p* = 0.632

**Table 5:** Odds ratio and regression coefficients.

Predictor	Estimate	SE	*p*	OR	CI 95%		Outcome	Classify
Time2	−44.133	0.354	0.000	0.000	−44.826	−43.440	C4	I
Time2	−44.759	0.354	0.000	0.000	−45.452	−44.066	TOTAL	I
Time2	−43.397	0.354	0.000	0.000	−44.090	−42.704	Q1	I
Time2	−43.530	0.740	0.000	0.000	−44.979	−42.080	Q4	I
Time2	−43.426	0.354	0.000	0.000	−44.119	−42.734	Q5	I
Time2	−43.596	0.451	0.000	0.000	−44.479	−42.712	Q11	I
Time2	−43.501	0.729	0.000	0.000	−44.930	−42.073	Q17	I
Time2	−45.482	0.375	0.000	0.000	−46.217	−44.748	C1	R
Time2	−3.861	0.669	0.000	0.021	−5.172	−2.549	C2	R
Time2	−45.602	0.383	0.000	0.000	−46.354	−44.851	C3	R
Time2	−4.516	1.066	0.000	0.011	−6.605	−2.427	C4	R
Time2	−45.368	0.368	0.000	0.000	−46.089	−44.648	TOTAL	R
Time2	−4.566	0.766	0.000	0.010	−6.067	−3.066	Q1	R
Time2	−2.249	0.516	0.000	0.105	−3.260	−1.239	Q2	R
Time2	−2.079	0.540	0.000	0.125	−3.138	−1.021	Q3	R
Time2	−4.043	0.686	0.000	0.018	−5.388	−2.698	Q4	R
Time2	−3.056	0.571	0.000	0.047	−4.175	−1.938	Q5	R
Time2	−45.608	0.354	0.000	0.000	−46.301	−44.915	Q6	R
Time2	−3.091	0.580	0.000	0.045	−4.227	−1.955	Q7	R
Time2	−2.412	0.532	0.000	0.090	−3.454	−1.369	Q8	R
Time2	−1.729	0.480	0.000	0.177	−2.670	−0.789	Q9	R
Time2	−2.828	0.564	0.000	0.059	−3.932	−1.723	Q10	R
Time2	−1.719	0.478	0.000	0.179	−2.655	−0.783	Q11	R
Time2	−2.610	0.534	0.000	0.074	−3.658	−1.563	Q12	R
Time2	−2.646	0.543	0.000	0.071	−3.709	−1.582	Q13	R
Time2	−2.828	0.564	0.000	0.059	−3.932	−1.723	Q14	R
Time2	−3.912	0.801	0.000	0.020	−5.482	−2.342	Q15	R
Time2	−3.050	0.678	0.000	0.047	−4.380	−1.721	Q17	R
Time2	−2.216	0.508	0.000	0.109	−3.211	−1.220	Q18	R
Time2	−2.539	0.611	0.000	0.079	−3.737	−1.341	Q19	R
Time2	−2.708	0.558	0.000	0.067	−3.801	−1.615	Q20	R
Time2	−2.325	0.670	0.001	0.098	−3.638	−1.013	Q15	I
Time2	−1.766	0.562	0.002	0.171	−2.868	−0.664	Q2	I
Time2	−3.328	1.060	0.002	0.036	−5.405	−1.251	Q16	R
Time2	−3.189	1.058	0.003	0.041	−5.263	−1.116	C1	I
Time2	−2.167	0.795	0.006	0.114	−3.725	−0.610	C3	I
Time2	−1.426	0.619	0.021	0.240	−2.639	−0.214	Q3	I

## Data Availability

The raw data supporting the conclusions of this article will be made available by the authors on request.
